# Wilderness Medicine Physician Education: How an Elective Can Spark a Fire

**DOI:** 10.7759/cureus.15317

**Published:** 2021-05-29

**Authors:** Andrew Belyea, Ari Fish, Lara Phillips

**Affiliations:** 1 Family Medicine, Dalhousie University, Charlottetown, CAN; 2 Emergency Medicine, Thomas Jefferson University, Philadelphia, USA; 3 Emergency Medicine, Thomas Jefferson University Hospital, Philadelphia, USA

**Keywords:** wilderness medicine, medical elective, resident, medical student, medical education

## Abstract

Background

Wilderness medicine (WM) electives offer an opportunity for medical trainees to learn an additional skillset outside of the traditional medical education curricula. Prior literature has yet to detail how participation in WM electives during medical training informs future training (i.e., master’s degree, fellowship) or career involvement in the field.

Methodology

A 25-question survey was completed by former participants of 25 WM electives based in the United States. Survey questions focused on the demographics, motivations, current involvement, and additional WM training among those who participated in WM electives. The survey was completed by 102 eligible participants.

Results

Of the 102 participants, 53% had been engaged with WM since completing their elective; 18% of the participants had completed additional formal training in WM (i.e., master’s degree, fellowship). Further, 95% of participants felt that the elective enhanced their resilience and critical thinking. Of those currently most involved in WM (n = 26), half (46%) were unsure about integrating WM into their careers prior to their elective. Among the uncertain yet highly engaged cohort, 98% cited the elective as the reason they stayed involved in WM.

Conclusions

These findings underscore the importance of WM electives in fostering interest among medical trainees in WM, and suggest that participation in WM electives may promote further involvement after medical school and residency.

## Introduction

Wilderness medicine (WM) broadly encompasses the delivery of medical care in austere and/or low-resource environments. Opportunities for medical students and resident training in wilderness medicine have multiplied in recent years [1]. Medical students and residents (hereafter referred to as “medical trainees”) have demonstrated a desire for exposure to and education in WM, with such experiences primarily being divided between nonelective experiences and elective courses [2,3]. Popular nonelective experiences include conferences, lectures, interest groups, weekend retreats, and wilderness races [4-6]. Many of these nonelective experiences offer an accessible opportunity to expose medical trainees to WM; however, because these programs are often short in duration, they typically provide limited exposure to the field.

For medical trainees seeking a more comprehensive WM experience, WM electives have become an increasingly popular option [2]. While there were only eight US-based WM electives in 2005, this number grew to at least 26 in 2014 [1]. Most electives either serve as broad introductions to various WM topics or specialize in a particular WM subfield (e.g., hyperbaric and dive medicine); because of their prehospital focus, they are particularly popular among emergency medicine (EM) trainees [7]. Exposure to WM via electives may spark an interest that promotes postresidency fellowship training, often after EM-based training programs, or career involvement in the field [7]. It is important to understand how WM electives influence participant career involvement, and studies have yet to examine such postelective involvement.

Prior research in a diverse set of medical specialties has demonstrated that medical school elective participation is a key factor in clarifying student decisions about future education and career choices in those fields [8]. Thus, the current study aims to: (i) outline professional demographics among past participants, (ii) identify how past participation in a WM elective by medical trainees influences their future involvement in the field of WM, and (iii) for those who are currently highly engaged in WM, understand the extent to which their elective experience influenced their current, active participation in WM.

## Materials and methods

The authors developed a survey to address these aims and included pilot testing with content experts in the field. A list of all medical school-sponsored WM electives in the United States (n = 25) was compiled using the Society for Academic Emergency Medicine’s (SAEM) online WM program directory. The decision to restrict our study to only medical school-based electives was made in order to examine the impact of elective programs on future physicians; many WM programs are not affiliated with medicals schools and do not require participants to be physician trainees. The survey was emailed to each of the WM elective directors (n = 25), who then distributed the survey to former elective participants. The survey consisted of 25 questions divided into three sections based on (i) demographics, (ii) elective benefits, and (iii) WM interest and involvement following an elective.

Inclusion criteria included having taken one of the 25 WM electives as a medical student or resident. Exclusion criteria included any participant who was a current medical student at the time of survey completion.

## Results

A total of 246 individuals received the survey, with 110 participants completing all 25 questions (45% response rate). Of the 110 participants, eight current medical students were excluded from the analysis, leaving 102 participants.

Attending physicians made up the greatest percentage of survey participants (41%), while 35% were early residents (postgraduate year [PGY]-1 or PGY-2), and 24% were late residents (PGY-3 through PGY-6). The most common specialty among participants was EM (45%), followed by family medicine (9%), and internal medicine (6%). Among all participants, 89% completed their WM electives during medical school, 5% during residency, and 6% during both medical school and residency (Table 1).

**Table 1:** Participant breakdown according to graduation year, training level at the time of elective, current level of training, and medical department. Current medical students were excluded.

		Count	%
Medical school graduation year	2015-2020	69	69
	Pre-2015	31	31
	No response	2	1
Training level at time of elective	Medical school	90	89
	Residency	5	5
	Both in medical school and residency	6	6
	No response	1	0
Current level of training	PGY1-PGY2	36	34
	PGY3-PGY6	24	24
	Attending	42	41
Department	Emergency medicine	46	45%
	Family medicine	9	9%
	Internal medicine	6	6%
	Psychiatry	5	5%
	General surgery	5	4%
	Orthopedic surgery	4	4%
	Plastic surgery	3	3%
	Pediatrics	3	3%
	Anesthesiology	3	3%
	Ophthalmology	2	2%
	Neurology	2	2%
	Vascular surgery	1	1%
	Undersea and hyperbarics	1	1%
	Sports medicine	1	1%
	Rheumatology	1	1%
	Pediatric surgery	1	1%
	Pediatric neurology	1	1%
	Oncology	1	1%
	Obstetrics and gynecology	1	1%
	Cardiothoracic surgery	1	1%
	Internal medicine and pediatrics	1	1%
	Gastroenterology	1	1%
	Family medicine and emergency medicine	1	1%
	None	1	1%

**Figure 1:**
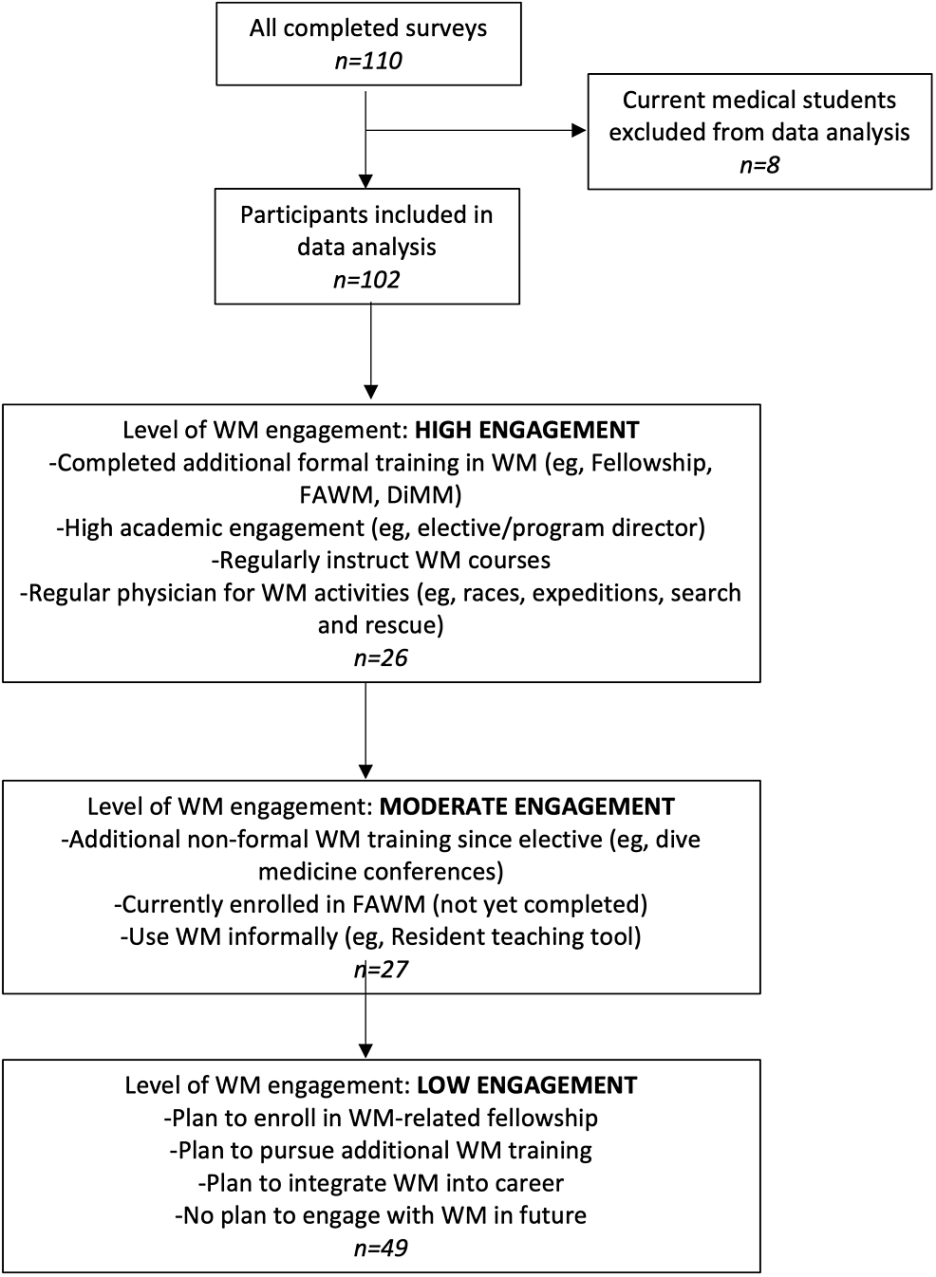
Inclusion and exclusion criteria. Included participants were assigned to one of the three groups based on WM engagement following their elective: high, moderate, and low engagement. FAWM: Fellowship of the Academy of Wilderness Medicine; DiMM: Diploma in Mountain Medicine; WM: wilderness medicine

Participants were divided into one of three subgroups for data analysis based on current involvement: high, moderate, and low engagement (Figure 1).

Overall, 25% of the total participants were in the high engagement subgroup (n = 26), 25% were in the moderate engagement subgroup (n = 27), and 48% were in the low engagement subgroup.

The majority (98%) of the participants reported that they benefited from participation in a WM elective. In total, 76% cited a new skillset and knowledge acquisition as the greatest perceived benefits of their elective, while 11% described “fun” as the greatest benefit. Further, 95% of the participants felt that the elective improved their resilience, critical thinking, and problem-solving. The following themes were cited as ways in which participants benefitted from WM electives: exposure to a new field, building confidence as a physician, developing knowledge and skills, gaining resource-limited training, networking, and hobby/personal interest fulfillment.

Among all participants, 78% reported an increased interest in WM following their elective. In total, 53% of participants had been engaged with WM since the completion of their elective; of those 53% of participants still involved, 47% cited their elective as a positive influencing factor in their decision to stay involved. Further, 80% of participants expressed that they were unsure about career WM involvement prior to their elective; of those uncertain participants, 37% cited the elective experience as the reason that they continued to stay involved.

A subgroup analysis allowed for a better understanding of high engagers’ participation in the field following the elective. Before the elective, 54% of high engagers planned on integrating WM into their career; the remaining 46% were uncertain whether they would stay involved. Of the uncertain participants, 98% cited the elective as the primary influencing factor that fueled their decision to stay involved. For those participants who were previously uncertain about future involvement, their elective experiences were sufficiently meaningful to ignite a desire for future WM engagement despite initial indifference.

In total, 60% of high engagers (18% of all participants) completed a WM fellowship or related master’s after their elective; among this supplemental training, 44% was in general WM, 8% in disaster medicine, 4% in global health, and 4% in hyperbaric medicine. Among those with supplemental WM training, 61% were EM physicians. This supports prior research suggesting that the majority of formal training programs in WM are linked to those who practice EM, though fellowship opportunities in other specialties are growing [7].

## Discussion

These results have implications for both trainees and curriculum developers in the fields of WM and EM. The vast majority of participants reported benefit from their elective, improving both subjective critical thinking and leadership skills along with objective low-resource medical management skills. The influence of electives appears to be long-lasting as many participants (53%) stay involved in WM in a variety of ways (teaching courses, fellowships, conference attendance, etc.) after they complete the elective.

Furthermore, our results demonstrate that there are a number of elective participants who, despite low initial interest, have their WM interest sparked by their elective course and subsequently become highly involved in the field. Overall, 80% of participants were unsure prior to their elective whether they would pursue long-term WM involvement; of that 80%, half pointed to their elective experience as the strongest motivating factor for their continued WM involvement. This observation is particularly salient among current high engagers, the most active current participants in WM: 96% of high engagers who were previously uncertain about future involvement indicated that their elective experience influenced them to pursue future WM participation. Of note, three high engagers who had no intention to pursue a career in WM prior to their elective eventually went on to complete additional fellowship training and are now WM fellowship program directors.

These findings should be considered in light of study limitations. As a retrospective study with self-reported outcomes, participants who remain invested in WM may have been more likely to complete the study survey. This may have resulted in an overestimation of the response rate. The time interval between elective completion and survey collection was not gathered because the authors believed making inferences on this data may have biased the analysis. As a cross-sectional study, we cannot comment on changes in WM engagement over time. Future research should consider follow-up questionnaires to monitor involvement in WM over time. Further, future studies should more closely track time since elective to establish a concrete temporal understanding of career involvement.

These data highlight the impact that WM electives can have on a participant’s career trajectory, even among those who were not initially considering further involvement in the field. This study illustrates that even those who are skeptical about future participation can have meaningful elective experiences which encourage sustained career involvement. For those individuals, the elective metaphorically sparked their fire of interest in WM.

## Conclusions

This study is the first to outline the professional demographics of past WM elective participants and examines how past participation in an elective influences future educational and career involvement in the field. These results demonstrate that most WM elective participants perceive benefit from their experience, with electives fostering interest for future involvement in the field. For many individuals, their elective experience is a strong motivating factor for sustained high engagement. Wilderness medicine electives encourage medical trainees to embrace low-resource medical training, serving to inspire those who may have never otherwise considered involvement in the field.

## Appendices

Questionnaire

Year of medical school graduation

Enter year _____

Current level of training

Medical Student year 3

Medical Student year 4

Resident PGY-1

Resident PGY-2

Resident PGY-3

Resident PGY-4

Resident PGY-5

Resident PGY-6

Resident PGY-7

Attending

Current training department

Emergency medicine

Family medicine & Emergency medicine

Family medicine

Anesthesiology

General surgery

Internal medicine

Obstetrics & Gynecology

Ophthalmology

Otolaryngology

Physical Medicine and Rehabilitation

Psychiatry

Pathology

Radiology

Urology

Other (please specify)_________________________

When did you do your wilderness medicine elective(s)?

Medical School

Residency

Both

What was your greatest motivating factor in taking a WM elective?

i.              Area of interest

ii.             Wanted a fun rotation

iii.            Wanted a challenge to try something new

iv.            Wanted more training in preparation for a career in WM

Before taking a WM elective, did you plan on a career in WM?

i.             Yes

ii.            No

iii.           Unsure

What was your largest perceived benefit from doing a WM elective?

i.              Fun

ii.             Exposure to a growing field

iii.            Expansion of my skillset and knowledgebase

iv.            Applicability to future WM work

v.             Minimal benefit

Do you feel that taking a WM elective had an impact on skills such as resilience, critical thinking, and problem-solving?

i.              Yes, taking a WM elective greatly developed my skills in these areas

ii.             Yes, taking a WM elective slightly improved my skills in these areas

iii.            Neutral, taking a WM elective had no impact on skills in these areas

iv.            No, taking a WM elective slightly worsened my skills in these areas

To what extent do you feel you benefited from or were disadvantaged by a WM elective?

i.               1-9 Likert Scale (1 = I was very disadvantaged, 5 = neutral, 9 = I benefited tremendously)

Did you reference taking a WM elective during your residency applications/interviews? (if yes, please explain by selecting “other”)

i.              Yes, every or almost every program

ii.             Sometimes, about half of programs

iii.            No, rarely spoke about elective

iv.            N/a, I was already a resident when I took the elective

v.             Other: ____

Did taking a WM elective impact your activities in residency?

i.               Yes

ii.              No

iii.            (optional) If yes, how so? _____

If you plan to integrate WM in your career (or have already), was that decision impacted by a WM elective?

i.              Yes I plan to pursue WM in my career; taking a WM elective influenced my decision

ii.             Yes I plan to pursue WM in my career; the WM elective had no impact

iii.            No I do NOT plan to pursue WM in my career; taking a WM elective influenced my decision

iv.            No I do NOT plan to pursue WM in my career; taking a WM elective had no impact

If you were involved in Wilderness Medicine in residency, in which field? (select all that apply)

i.              Dive medicine

ii.             Mountain and avalanche medicine and altitude illness

iii.            Tropical medicine

iv.            Expedition medicine

v.             Disaster medicine

vi.            Humanitarian medicine

vii.           Global health

viii.          Wilderness medicine education

ix.            Wilderness medicine research

x.             Search and rescue

xi.            Race medicine

xii.           Yes, other (please specify):

xiii.          N/A, I was not involved in WM in residency

Have you attended WM conference(s) since your WM elective?

i.              Yes, I try to attend at least one WM conference annually

ii.             Yes, I attend one every few years

iii.            Yes, I attended a single conference after the elective

iv.            No, I do not attend WM conferences

How did your elective influence your decision to attend WM conferences?

i.              The elective encouraged me to attend WM conferences

ii.             The elective did not play a role in whether or not I attend conferences

iii.            The elective discouraged me from attending WM conferences

Have you ever presented at WM conferences?

i.              Yes, one time

ii.             Yes, 2-4 times

iii.            Yes, >5 times

iv.            No

Do you have any WM publications, excluding conference presentations? (e.g., in magazines, peer-reviewed journals, blogs, etc.)

i.              Yes, one

ii.             Yes, 2-4

iii.            Yes, >5

iv.            No

Did you complete a Wilderness Medicine Fellowship or related fellowship (non-FAWM)?

i.              No

ii.             Yes, in Wilderness Medicine

iii.            Yes, in Hyperbaric Medicine

iv.            Yes, in Disaster Medicine

v.             Yes, in Climate Change

vi.            Yes, in Global Health

vii.           Yes, in Extreme Environmental Medicine

viii.          Yes, in Other (specify)

How did your WM elective influence your decision to pursue the Fellowship of the Academy of Wilderness Medicine (FAWM)?

i.              I had already received the fellowship before my WM elective

ii.             It positively influenced me, and I plan to enroll in the future

iii.            It positively influenced me, and I am currently enrolled

iv.            It positively influenced me, and I have completed it since my elective

v.             It neither encouraged nor discouraged me from pursuing the FAWM

vi.            It negatively influenced me. I had been considering enrolling, but the WM elective changed my mind.

vii.           It negatively influenced me by confirming WM was not something I was interested in.

How has your interest in WM been influenced by your elective?

i.               The elective greatly increased my interest in WM

ii.              The elective moderately increased my interest in WM

iii.             The elective has not changed my interest in WM

iv.             The elective moderately decreased my interest in WM

v.              The elective greatly decreased my interest in WM

If you wish to describe anything regarding your WM elective experience that was not captured in the questionnaire, please do so here.

If you are currently engaged with WM, please describe so here (e.g. teaching WM courses, serving as medical guide on expeditions, significant research engagement, regularly attending conferences).
